# Supplementary material for: Empirical analysis of vegetation dynamics and the possibility of a catastrophic desertification transition
Source: PLoS One. 2017 Dec 20;12(12):e0189058. doi: 10.1371/journal.pone.0189058 (PMC5737887; doi:10.1371/journal.pone.0189058)
Supplement: S1 Appendix — (PDF) [file pone.0189058.s001.pdf]

S1 Appendix for 'Empirical analysis of vegetation dynamics and the possibility of a catastrophic desertification transition'

Haim Weissmann[1*], Rafi Kent[2], Yaron Michael[2], Nadav M. Shnerb[1],

1 Department of Physics, Bar-Ilan University, Ramat-Gan IL52900, Israel.
2 Department of Geography and Environment, Bar-Ilan University, Ramat-Gan IL52900, Israel.

* wchaimw@gmail.com

S1 Appendix.

1.1 Correlation coefficient and number of pixels

Table S1 shows the number of pixels for each set of rainfall lines and the correlation coefficient between the years 1999-2002 and 2002-2015. In general the correlation coefficients are between 0.3 and 0.7, meaning that there is a substantial overlap between the local measures of vegetation cover, still there are also substantial modifications so the tracking of clusters and local cover is meaningful.

1.2 Spatial response curve

The Figs S1 and S2 below provide supplemental information to the data presented in Fig 2C of the main paper, that refers only to the region between 450 and 500 mm/y. Here the results are presented for all rainfall lines. For each rainfall regime, the blue open circles are the data, i.e., the average growth/shrink in size of a cluster of n pixels ($\log_{10}(n)$ is the x-axis). To show the general trend we have smoothed the data using the Matlab smooth algorithm (black full line, span parameter: 0.9, method: loess) [1]. In most cases one sees a clear positive feedback for small clusters but for large clusters the line curves down. A fit of the data to a parabola (full red line) using the function "glm" in R environment [2] is very similar to the smooth curve (the Student t-test with the data gives (unless otherwise noted) p-values below 5%) in most cases and indicates better the decay in the growth rate for large clusters. Green lines appear when the p-value for a parabola was too big and we have used linear regression instead.

1.3 Local response curve

The Figs S3-S11 below are a supplement for Fig 2B of the main text. The local response is presented for different rainfall lines. Given the vegetation density at a pixel, ρ_{ij}^t (in 1999, say) and the density at the same pixel in the next census (say, 2002), ρ_{ij}^{t+1}, the local response is defined as:

$$LRC_{ij} = \frac{\alpha \rho_{ij}^{t+1} - \rho_{ij}^t}{\rho_{ij}^t} \quad (1)$$

where

$$\alpha \equiv \frac{\langle \rho^t \rangle}{\langle \rho^{t+1} \rangle},$$

and $\langle \rho^t \rangle$ is the average (over all the rainfall line area) vegetation density in the first census. In the panels below we present LRC vs. $\rho^t_{ij}/\langle \rho^t \rangle$ (errorbars in S4-S5 with $STD = 1$, see also Fig S12). The response is clearly negative, except for the years 99-02 for rainlines 850-900, where one may notice weak positive feedback.

1.4 Histograms

As a complementary for Fig 4 of the main text, here we show the histograms of vegetation density values for different rainfall lines.

References

1. MATLAB. *version 8.6.0.267246 (R2015b)*. Natick, Massachusetts, 2015.

2. R Core Team. *R: A Language and Environment for Statistical Computing*. R Foundation for Statistical Computing, Vienna, Austria, 2016.
