# Supplementary figures and images for: Empirical analysis of vegetation dynamics and the possibility of a catastrophic desertification transition
Source: PLoS One. 2017 Dec 20;12(12):e0189058. doi: 10.1371/journal.pone.0189058 (PMC5737887; doi:10.1371/journal.pone.0189058)

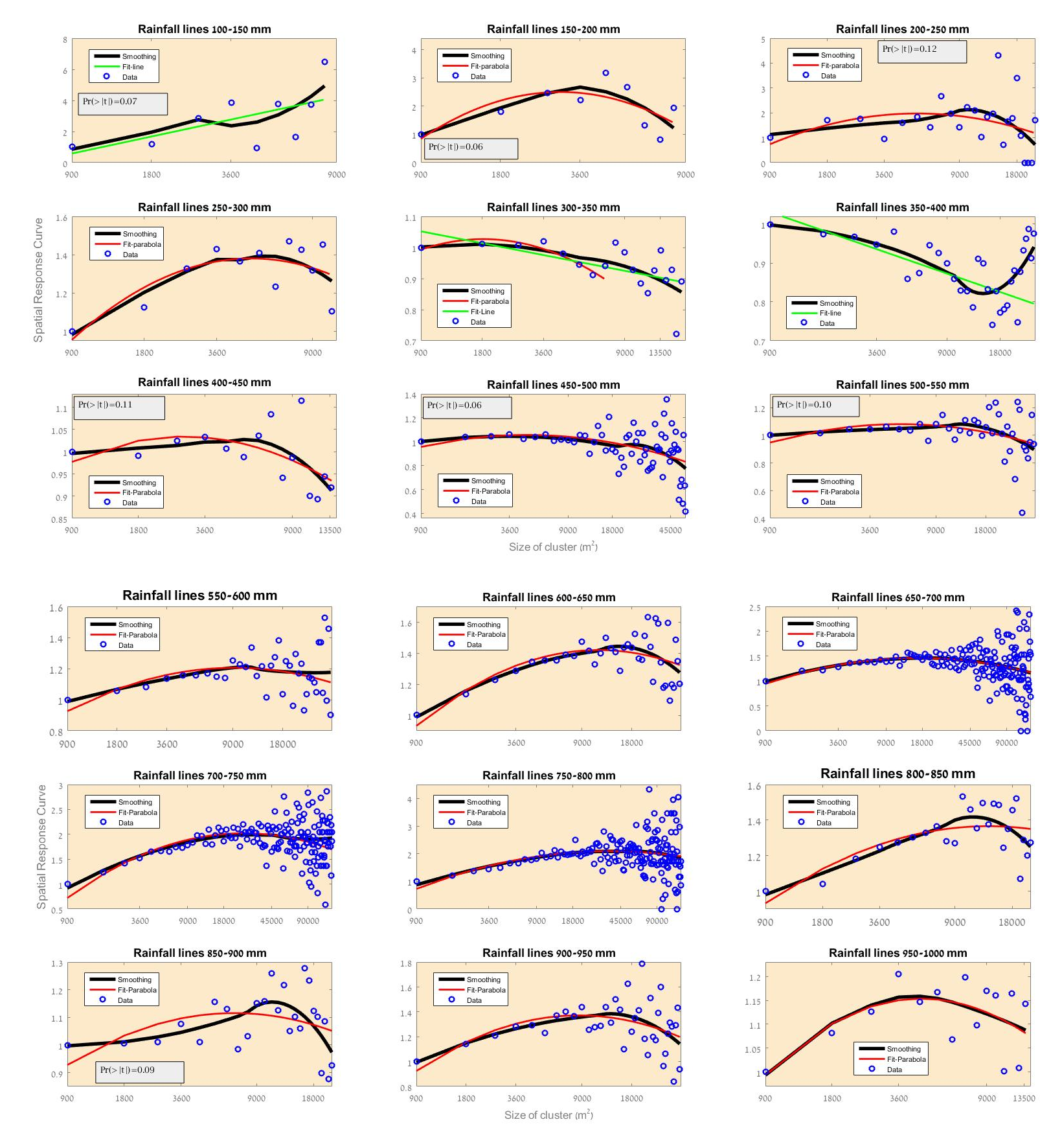

Supplement: S1 Fig — (TIFF) [file pone.0189058.s002.tiff]

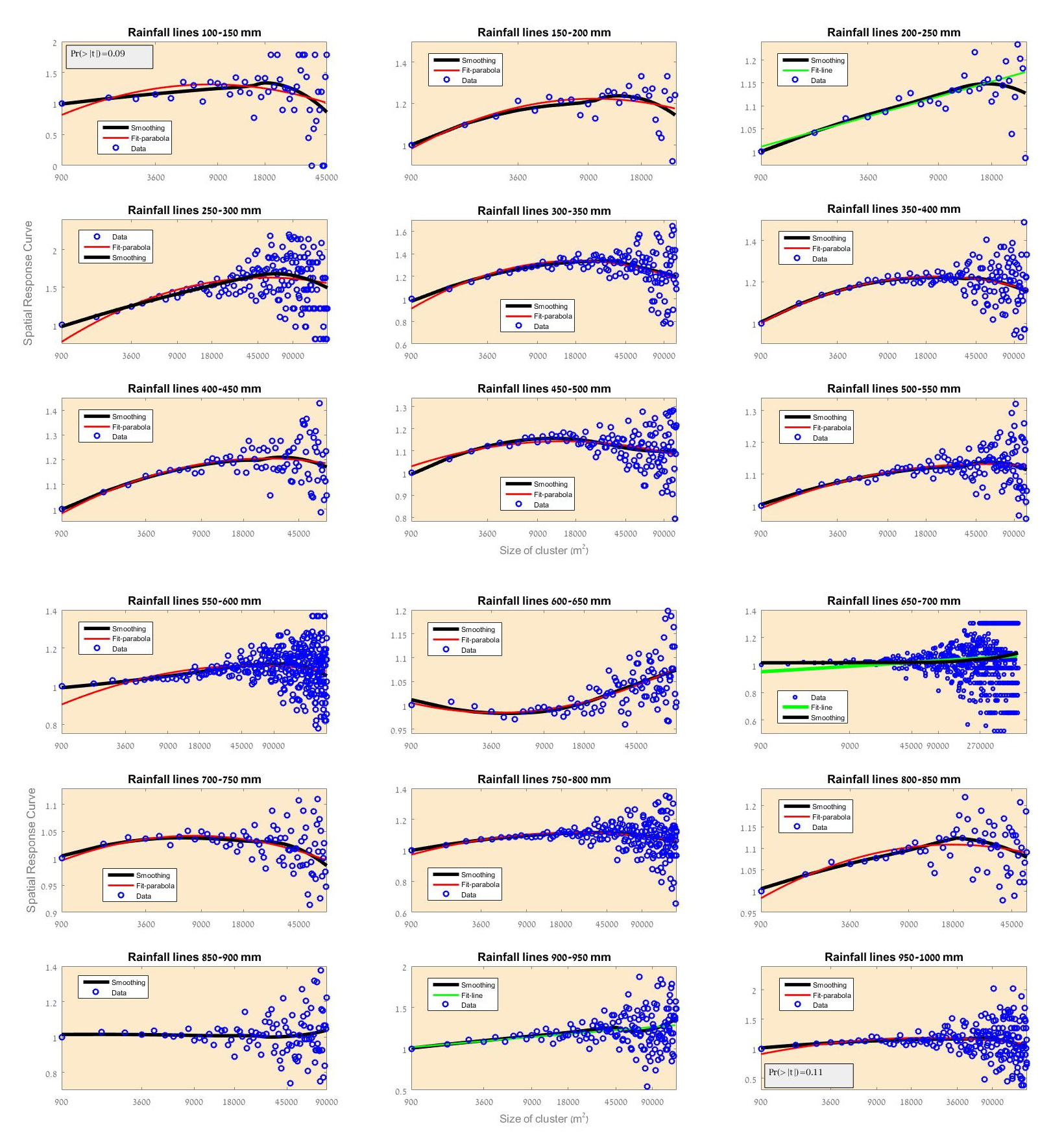

Supplement: S2 Fig — (TIFF) [file pone.0189058.s003.tiff]

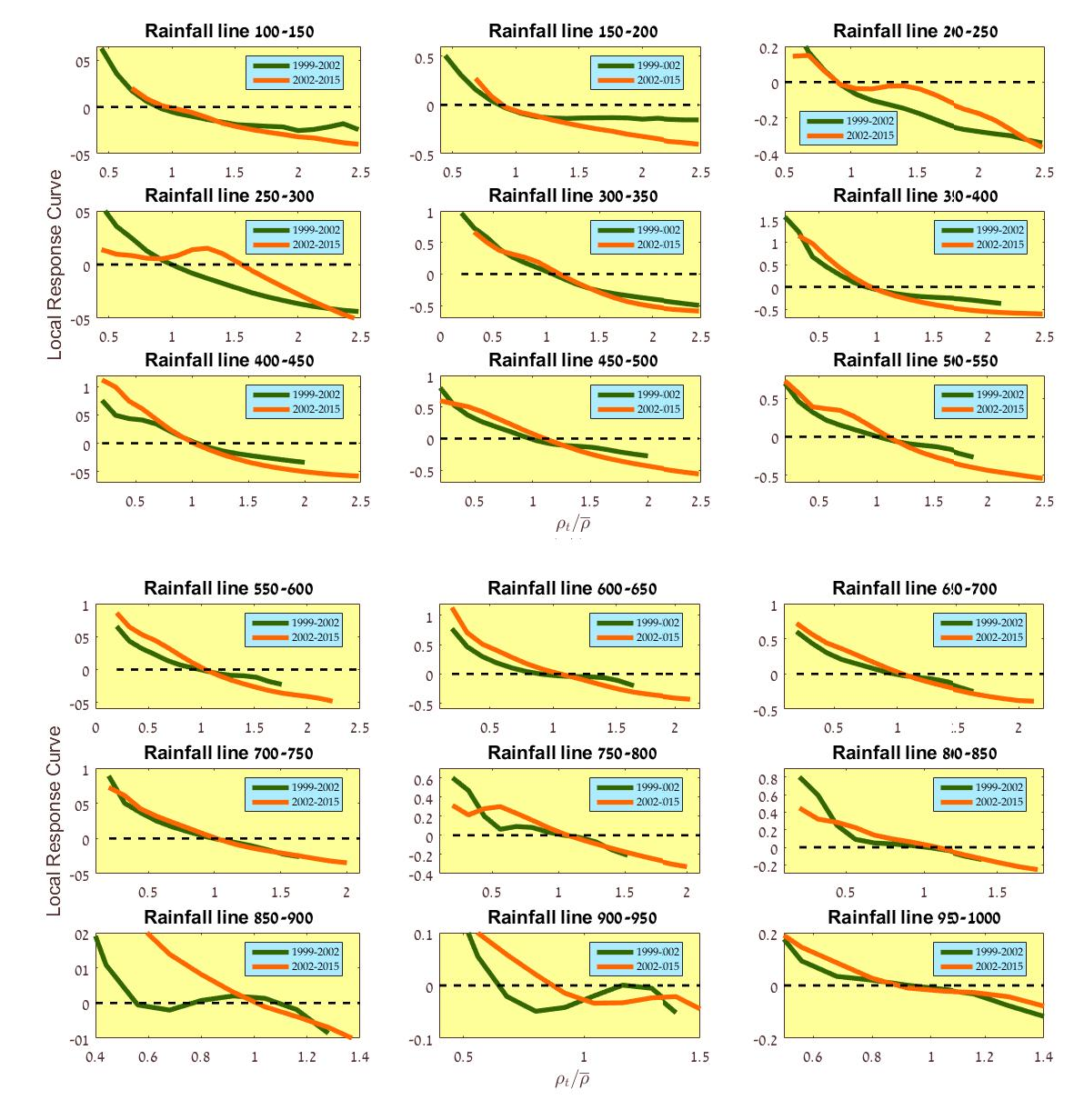

Supplement: S3 Fig — (TIFF) [file pone.0189058.s004.tiff]

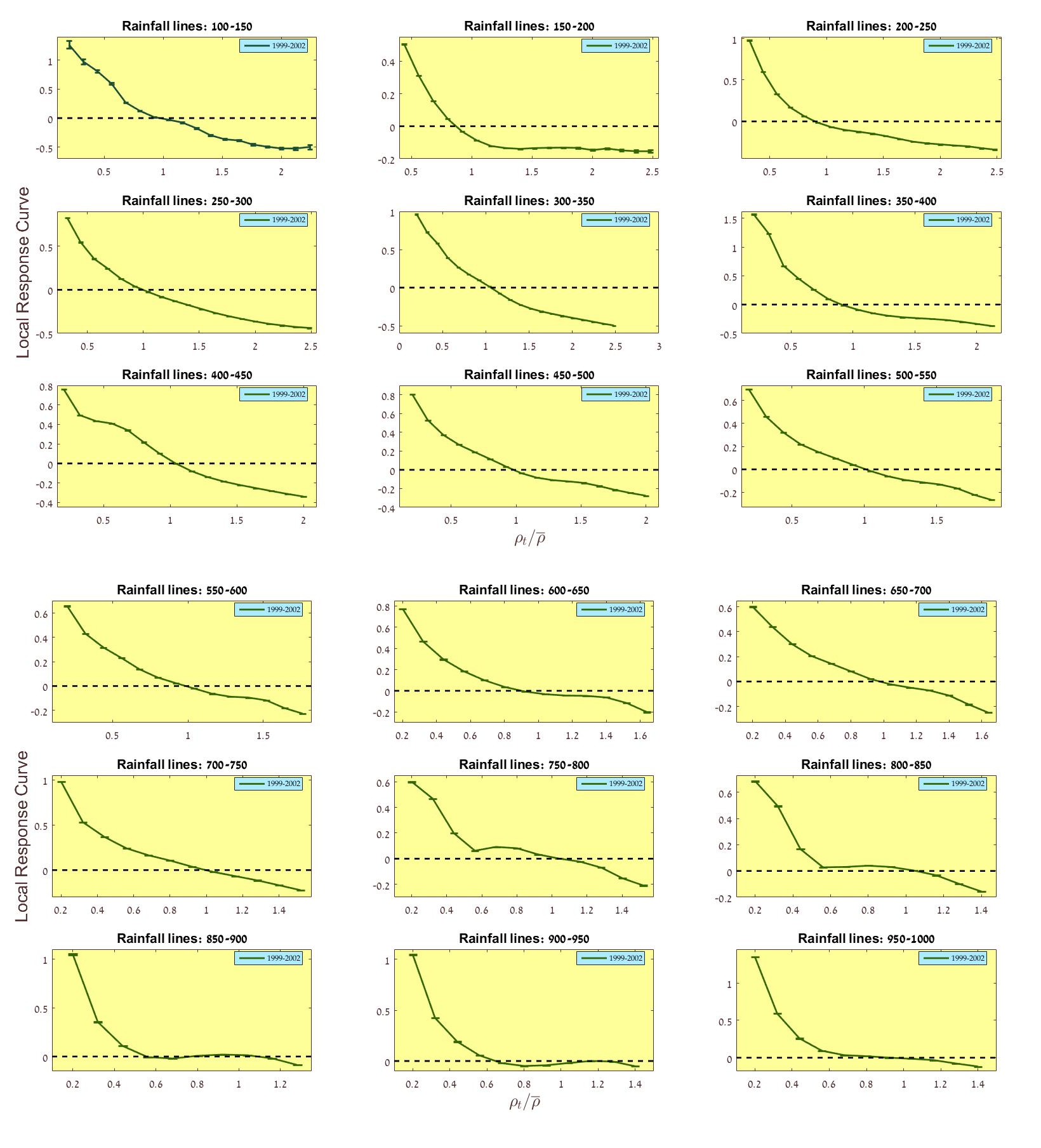

Supplement: S4 Fig — (TIFF) [file pone.0189058.s005.tiff]

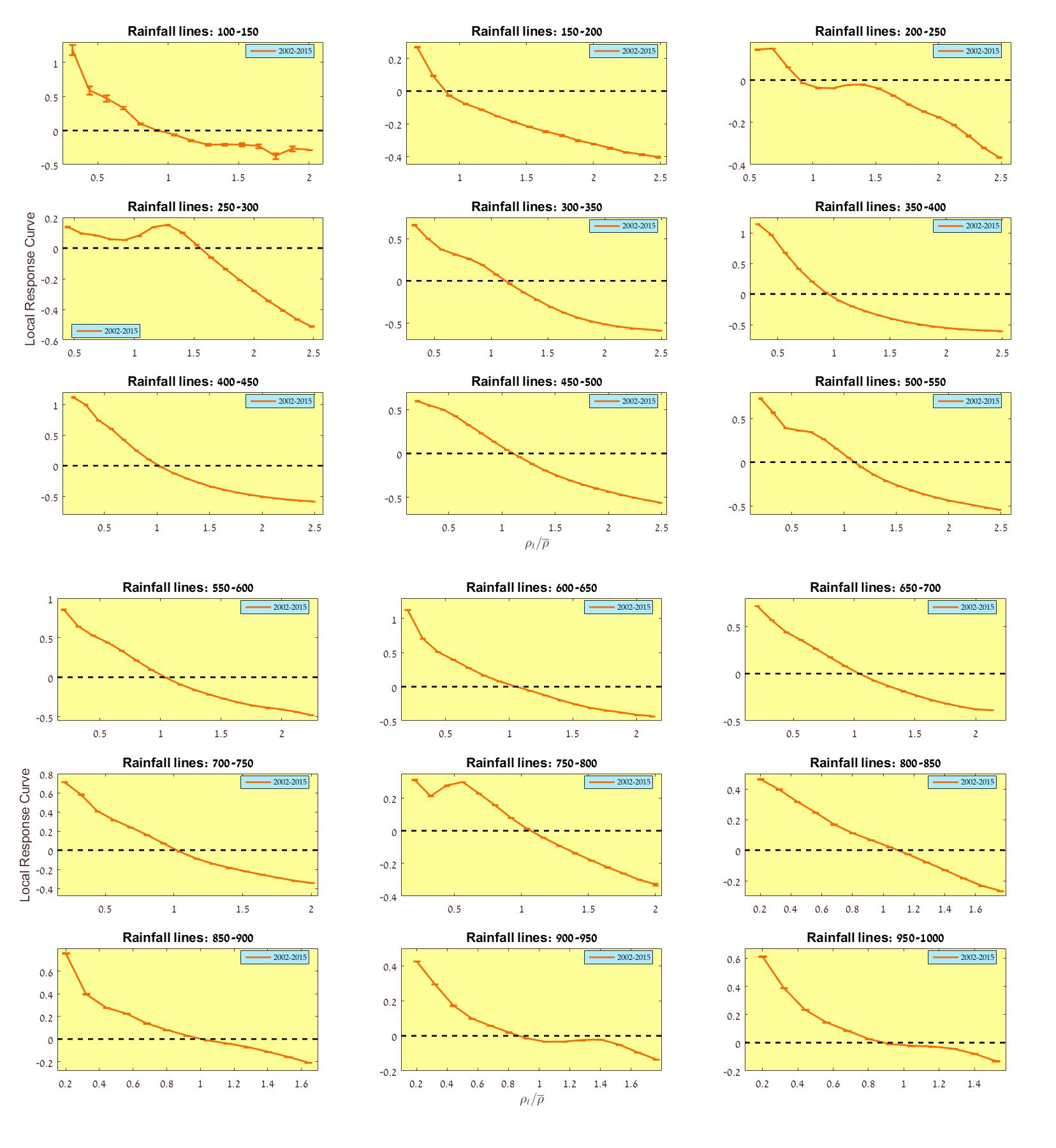

Supplement: S5 Fig — (TIFF) [file pone.0189058.s006.tiff]

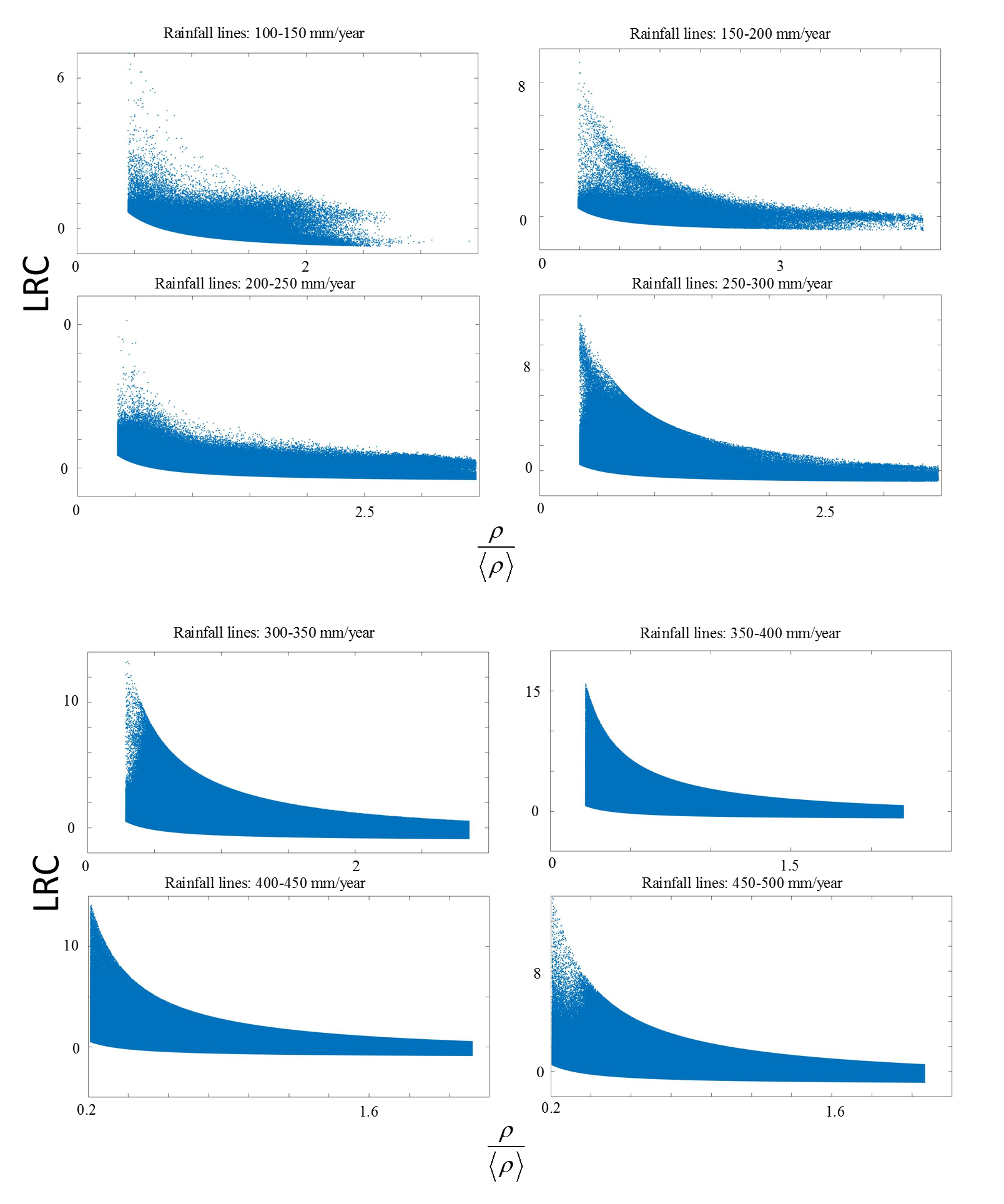

Supplement: S6 Fig — (TIFF) [file pone.0189058.s007.tiff]

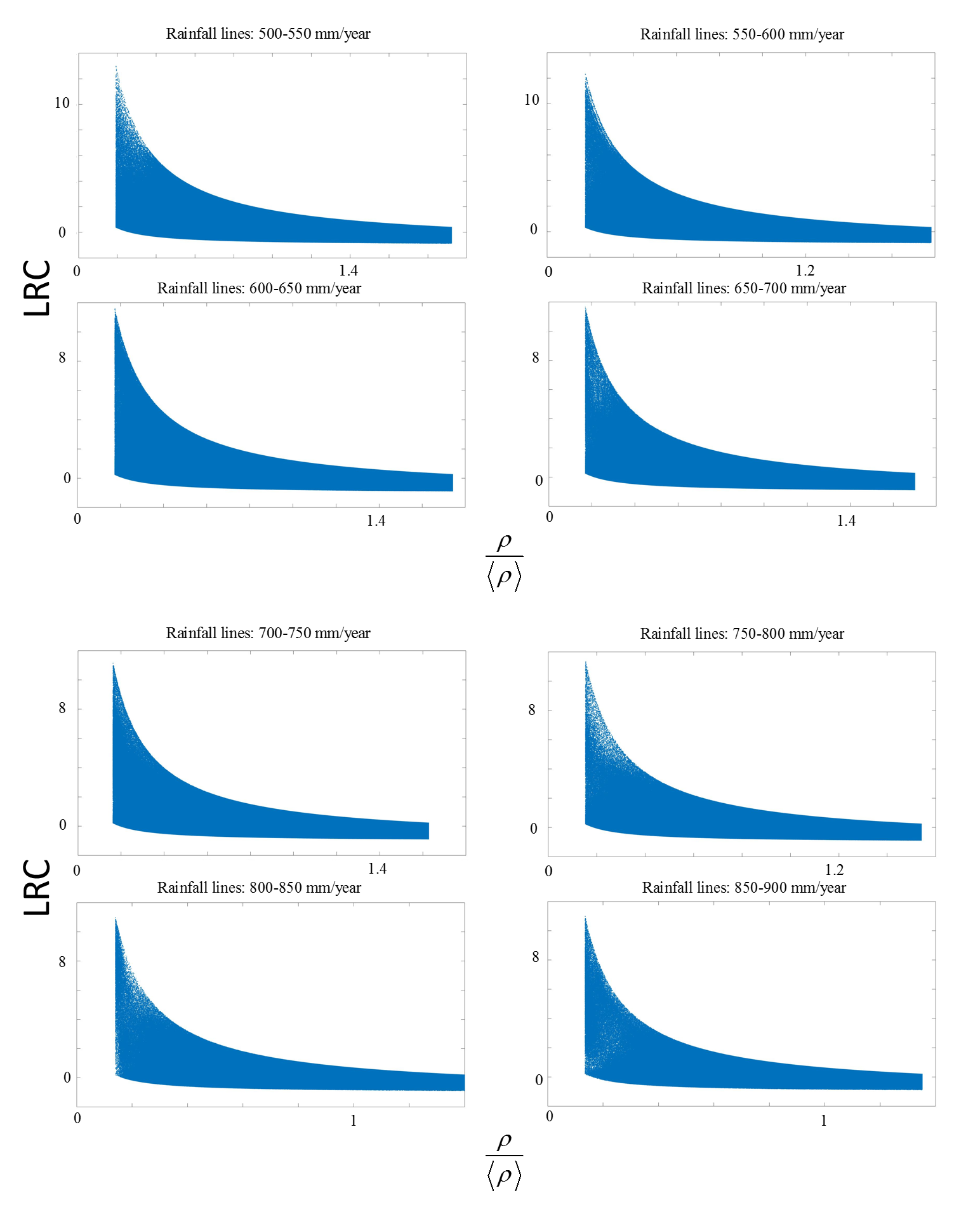

Supplement: S7 Fig — (TIFF) [file pone.0189058.s008.tiff]

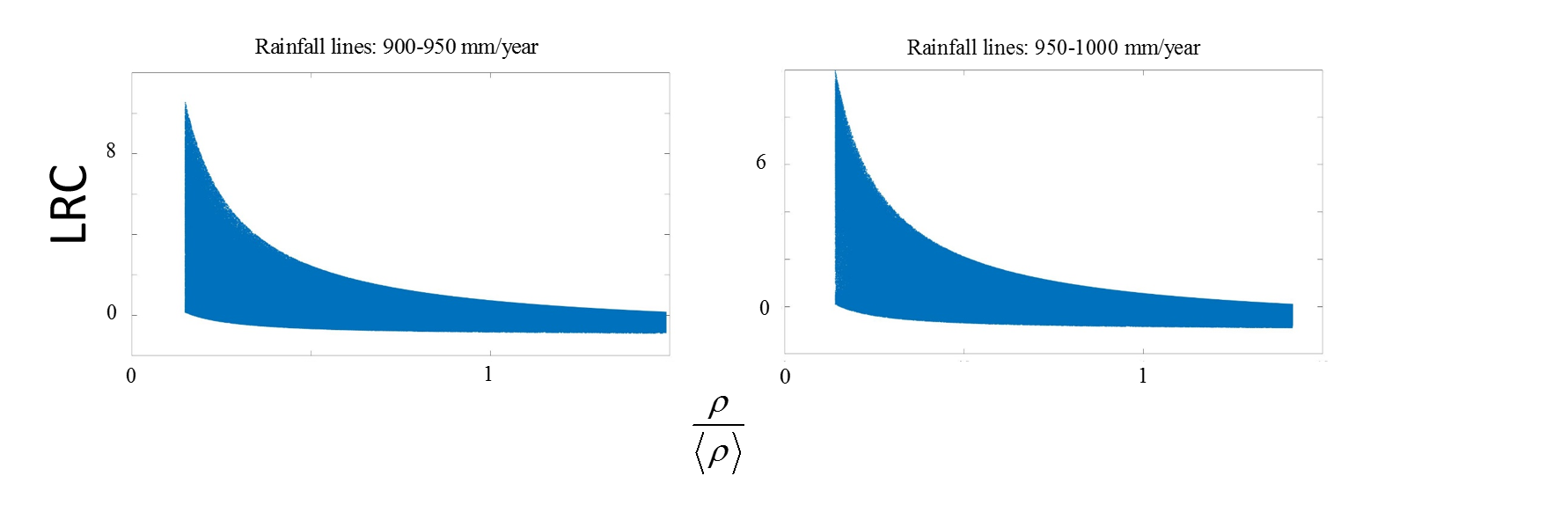

Supplement: S8 Fig — (TIFF) [file pone.0189058.s009.tiff]

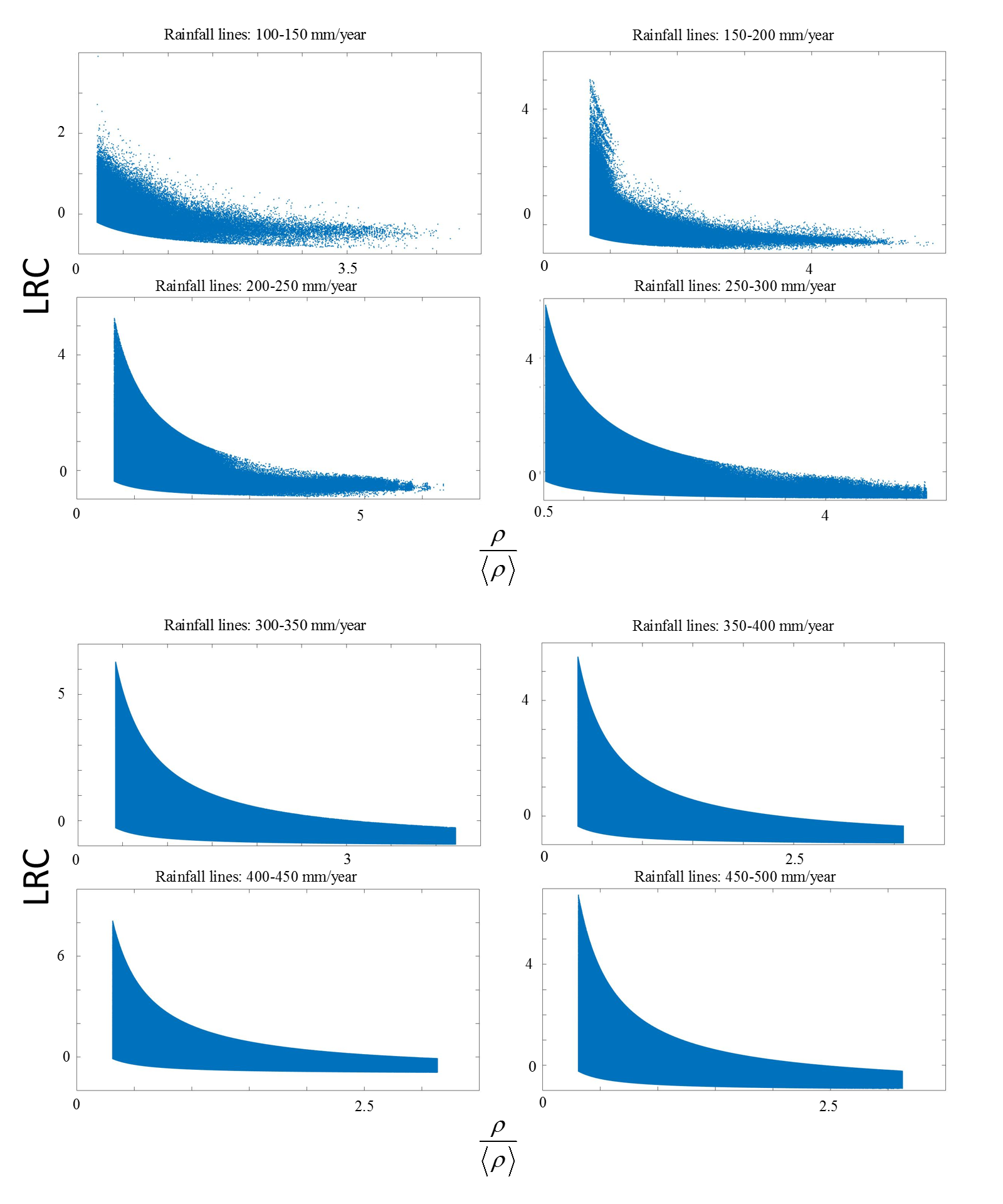

Supplement: S9 Fig — (TIFF) [file pone.0189058.s010.tiff]

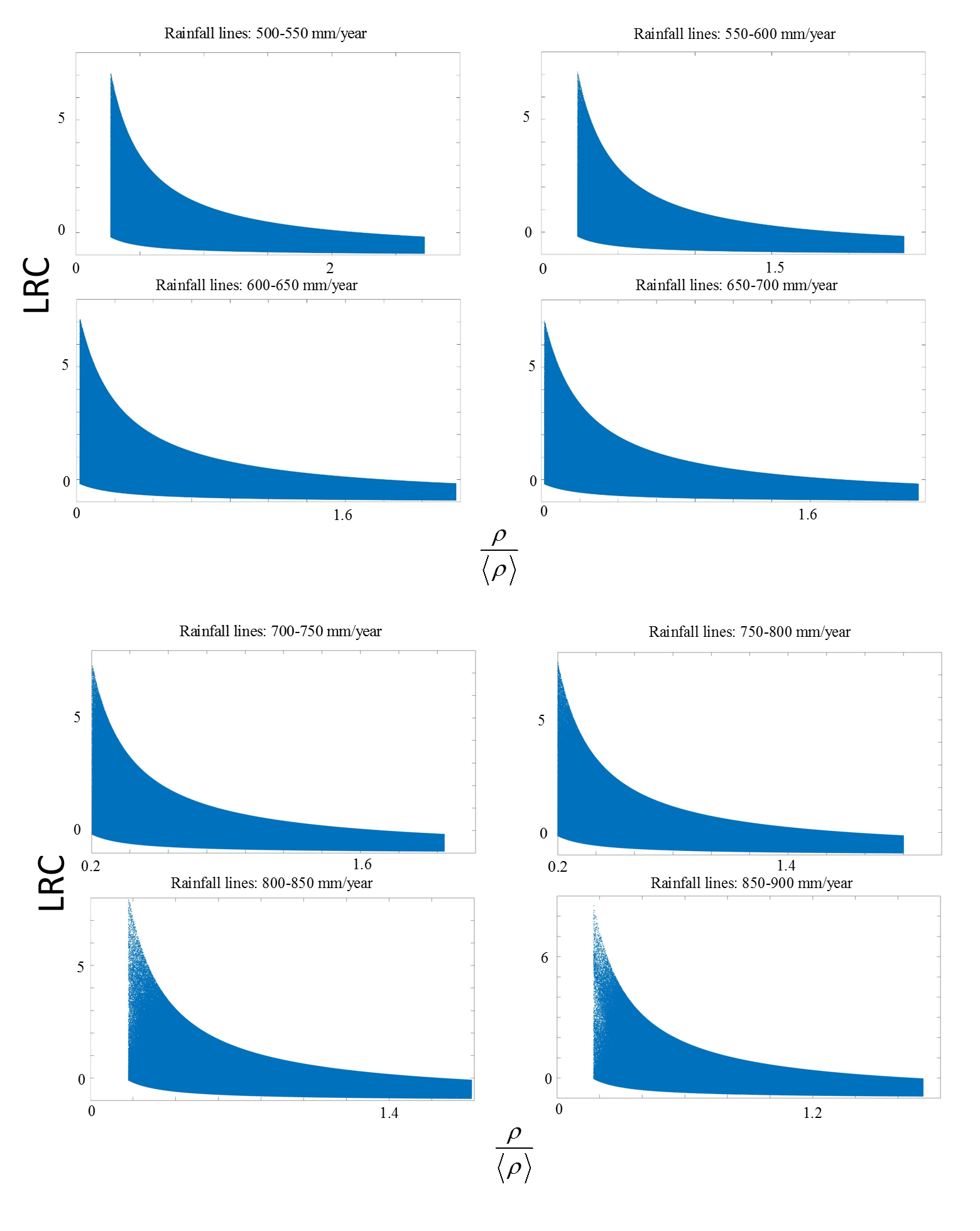

Supplement: S10 Fig — (TIFF) [file pone.0189058.s011.tiff]

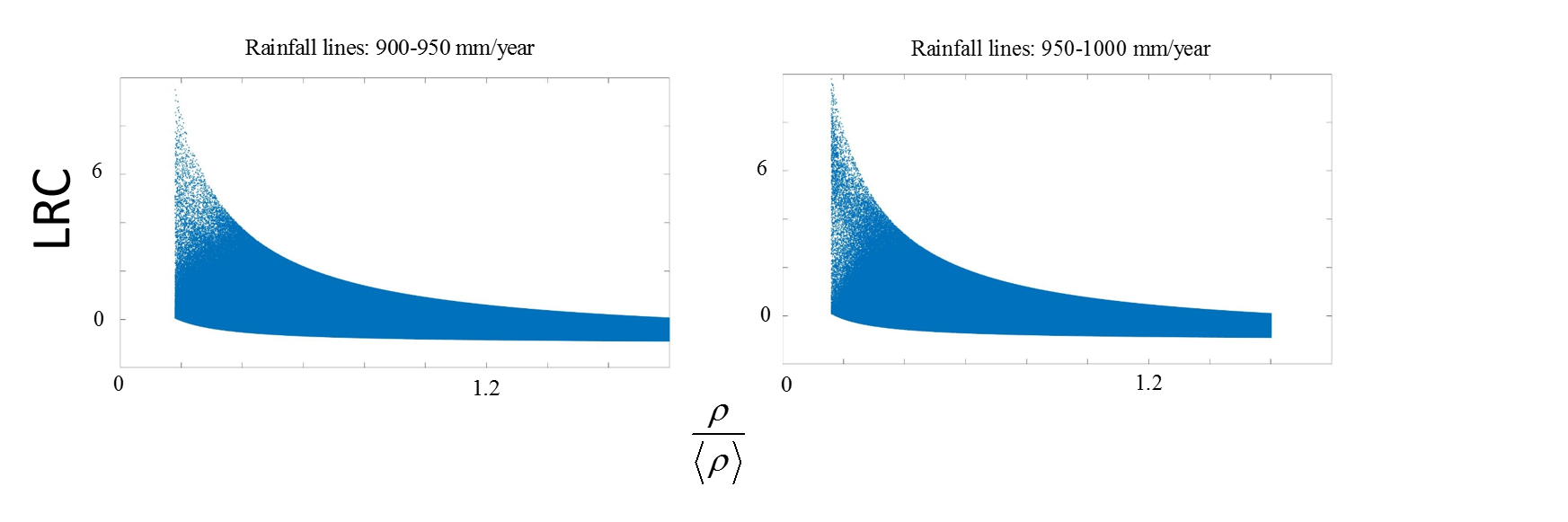

Supplement: S11 Fig — (TIFF) [file pone.0189058.s012.tiff]

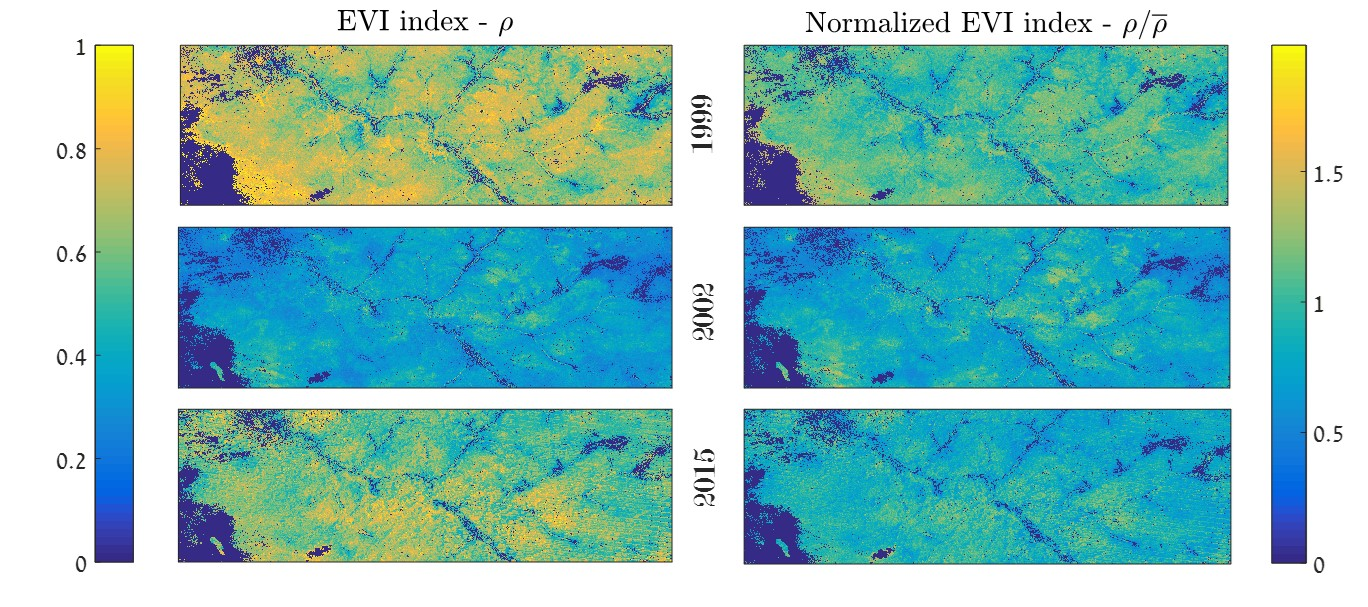

Supplement: S12 Fig — A sample of 2250km2 from the studied area (latitude: 14.7° − 15.02° and longitude: 19.3° − 19.6°). (TIFF) [file pone.0189058.s013.tiff]

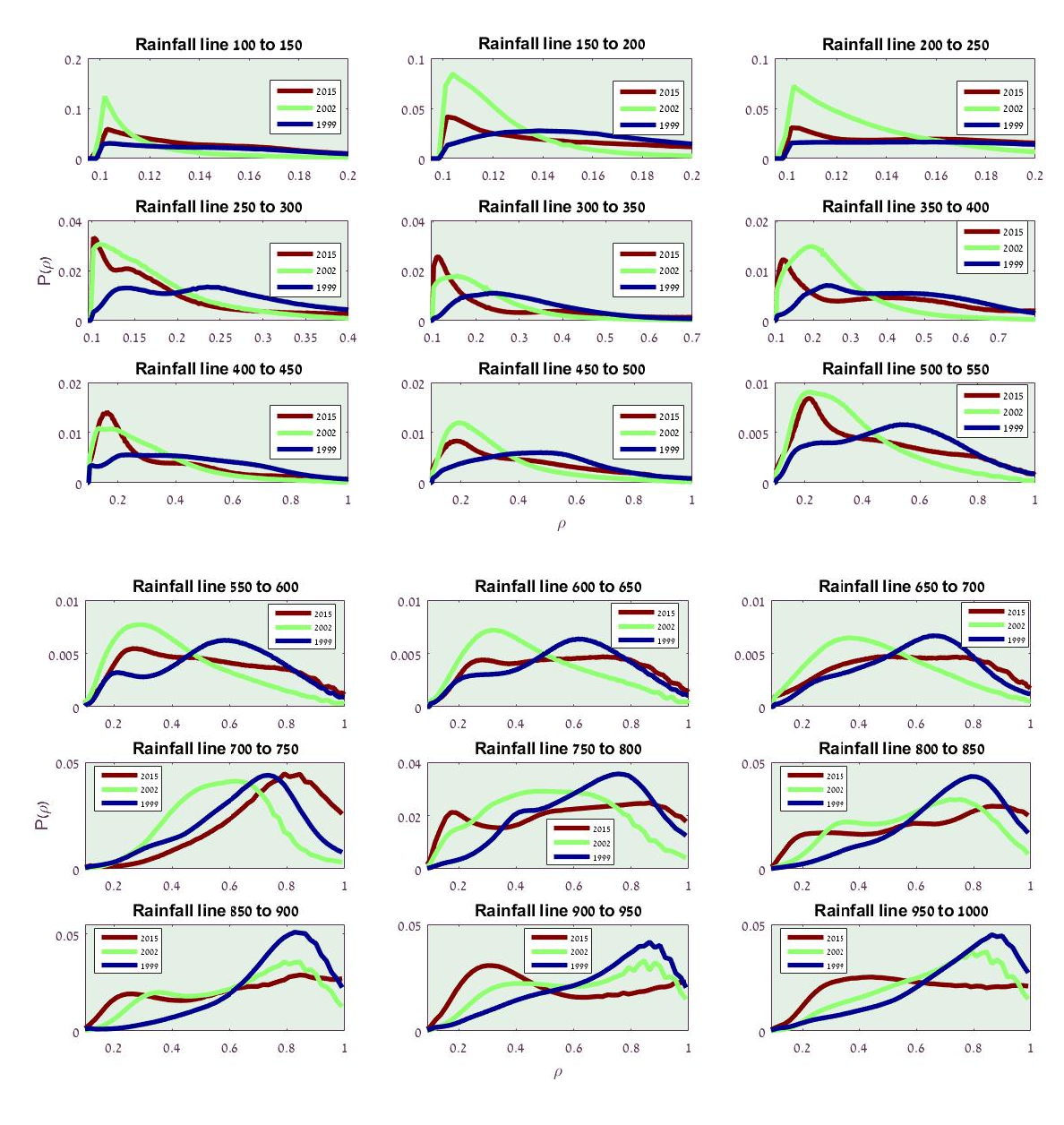

Supplement: S13 Fig — (TIFF) [file pone.0189058.s014.tiff]

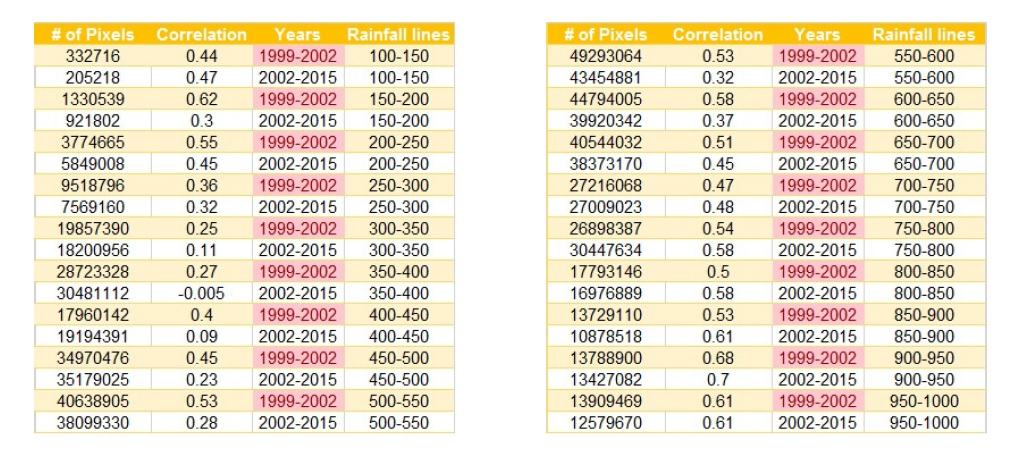

Supplement: S1 Table — (TIFF) [file pone.0189058.s015.tiff]
